# Dose-dependent modulation of microglia activation in rats after penetrating traumatic brain injury (pTBI) by transplanted human neural stem cells

**DOI:** 10.1371/journal.pone.0285633

**Published:** 2023-05-16

**Authors:** MaryLourdes Andreu, Nathalie Matti, Helen M. Bramlett, Yan Shi, Shyam Gajavelli, W. Dalton Dietrich

**Affiliations:** 1 Miami Project to Cure Paralysis, University of Miami Miller School of Medicine, Miami, Florida, United States of America; 2 Division of Pathology, Department of Clinical Sciences Lund, Lund University, Lund, Sweden; 3 Department of Neurological Surgery, University of Miami Miller School of Medicine, Miami, Florida, United States of America; 4 Bruce W. Carter Department of Veterans Affairs Medical Center, Miami, Florida, United States of America; Louisiana State University Health, Shreveport, UNITED STATES

## Abstract

Traumatic brain injury (TBI) often results in long-lasting patterns of neurological deficits including motor, sensory, and cognitive abnormalities. Cranial gunshot survivors are among the most disabled TBI patients and face a lifetime of disability with no approved strategies to protect or repair the brain after injury. Recent studies using a model of penetrating TBI (pTBI) have reported that human neural stem cells (hNSCs) transplantation can lead to dose and location-dependent neuroprotection. Evidence for regional patterns of microglial activation has also been reported after pTBI with evidence for microglial cell death by pyroptosis. Because of the importance of injury-induced microglial activation in the pathogenesis of TBI, we tested the hypothesis that dose-dependent hNSC mediated neuroprotection after pTBI was associated with reduced microglial activation in pericontusional cortical areas. To test this hypothesis, quantitative microglial/macrophage Iba1 immunohistochemistry and Sholl analysis was conducted to investigate the arborization patterns using four experimental groups including, (i) Sham operated (no injury) + low dose (0.16 million cells/rat), (ii) pTBI + vehicle (no cells), (iii) pTBI + low dose hNSCs (0.16 million/rat), and (iv) pTBI + high dose hNSCs (1.6 million cells/rat). At 3 months post-transplantation (transplants at one week after pTBI), the total number of intersections was significantly reduced in vehicle treated pTBI animals versus sham operated controls indicating increased microglia/macrophage activation. In contrast, hNSC transplantation led to a dose-dependent increase in the number of intersections compared to pTBI vehicle indicating less microglia/macrophage activation. The peak of Sholl intersections at 1 μm from the center of the microglia/macrophages ranged from ~6,500–14,000 intersections for sham operated, ~250–500 intersections for pTBI vehicle, ~550–1,000 intersections for pTBI low dose, and ~2,500–7,500 intersections for pTBI high dose. Plotting data along the rostrocaudal axis also showed that pericontusional cortical areas protected by hNSC transplantation had increased intersections compared to nontreated pTBI animals. These studies using a non-biased Sholl analysis demonstrated a dose-dependent reduction in inflammatory cell activation that may be associated with a neuroprotective effect driven by the cellular transplant in perilesional regions after pTBI.

## Introduction

Traumatic brain injury (TBI) often leads to a range of severe disabilities and loss of function in patients. In the United States, approximately 1.7 million individuals sustain a TBI annually [1]. The causes of TBI may include, but are not limited to, falls, assault, motor-vehicle accidents, or being struck by or against an object [1]. Patients with gunshot wounds that produce penetrating TBI (pTBI) have a higher mortality in some cases [2, 3]. Currently, surgical therapies are an option for improving survival after pTBI combined with intensive care management including neuromonitoring [4].

The rodent model of penetrating traumatic brain injury (pTBI) [5–7] recapitulates neuropathological features such as progressive tissue loss and failure of numerous agents that also failed in clinical trials [4]. Progressive tissue loss after pTBI does not resolve [8] and recent studies have reported that increased and prolonged inflammasome activation in cortical and subcortical regions by immunoblot and immunohistochemical approaches [9, 10]. It is known that activated inflammatory cells in pericontusional areas after TBI are associated with the production of various proinflammatory mediators representing an important secondary injury mechanism and target for therapeutic interventions [11]. For example, Lee and colleagues [10] reported persistent microglial activation using unbiased stereological approaches up to 12 weeks after pTBI. In the steady scavenging state, microglia normally have processes that extend and retrack to monitor and scan the local environment [12]. However, under various pathological conditions, these thin and ramified processes are reduced and appear thicker indicating various levels of activation. In addition, evidence for pyroptotic cell death in neurons and activated microglia located in the pTBI penumbra was observed and associated with lesion expansion over time [9, 10]. Thus, strategies that target posttraumatic inflammatory mechanisms are currently being tested in both animal and clinical studies [13–18].

The potential beneficial effects of neural stem cell (NSC) transplantation has been examined in various models of brain and spinal cord injury [4]. In addition to having the capacity to replace lost neurons, NSCs can stimulate recovery by repairing damaged neural tissues, stabilizing TBI lesion, and modulating neuroinflammation. Using a rat model of pTBI, Hu and colleagues [19] reported evidence for neuroprotection following transplantation of human NSC transplantation when the cells were injected into perilesional regions. In that study, hNSC transplanted at 1 week in the tissue surrounding the lesion but not within the lesion area leads to significant cortical sparing compared to pTBI animals treated with vehicle. Based on these published data we hypothesized that hNSC transplantation in the pTBI penumbra would modulate patterns of injury-induced microglia/macrophage activation and therefore participate in the hNSC-dependent neuroprotective response [10, 19].

To obtain structural evidence for hNSC transplantation altering microglial activation, we objectively quantified regional patterns of activation using the Sholl analysis protocol to evaluate changes in microglial branching complexity by determining numbers of intersections at various distances from the cell soma [20, 21]. This unbiased morphological evaluation was assessed in four brain regions including the ipsilateral and contralateral cerebral cortices, and ipsilateral and contralateral dentate gyrus. We tested two dosages of hNSCs to evaluate dose-response effects combined with Iba1 immunohistochemistry.

## Methods

### Animals

Animal procedures adhered to the National Institute of Health (NIH) guidelines for the Care and Use of Laboratory Animals and the Animal Research: Reporting In Vivo Experiments (ARRIVE). The procedures were approved by the U.S. Army Medical Research and Material Command (USAMRMC) Animal and Use Committee (ACURO), and the University of Miami’s Institutional Animal Care and Use Committees (IACUC). In this experiment, male Sprague Dawley rats (8–10 weeks old; *N* = 32) weighing approximately 280 g were used. Although both human males and females sustain TBIs, for this investigation male rats were used since human males have a higher risk of TBI compared to females. The highest human male-to-female ratio of TBI typically occurs in adolescence and young adulthood [22]. Therefore, the male Sprague Dawley rats utilized parallel a similar demographic to those most susceptible to TBI. Rats were randomly assigned to each experimental group: (i) sham *n* = 4 (no pTBI + 0.16 million hNSCs), (ii) vehicle *n* = 9 (pTBI + no hNSCs), (iii) low dose *n* = 9 (pTBI + 0.16 million hNSCs), and (iv) high dose *n* = 10 (pTBI + 1.6 million hNSCs) (Table 1).

**Table 1:** Descriptive data of dosage quantity, number of animals per group, and number of animals analyzed are listed.

Group	Sham	Vehicle	Low dose	High dose
Injury	No	Yes	Yes	Yes
hNSCs quantity	0.16 million	0	0.16 million	1.6 million
Number of animals	4	9	9	10
Microglia Analysis	4	9	9	10

### Power analysis

For this study, the power analysis was based on pilot and previously published studies [8, 19, 23]. The G*Power 3.1 with type 1 error α at 0.05, power (1- type II error β) set to 0.8 and estimated effect size (Cohen’s d) d = 0.66 to calculate the sample size for lesion as a main outcome. The smaller sham sample size was deemed sufficient based on (i) previous publication with regards to microglia [10] (ii) minimal transplant engraftment and (iii) lesion size. The injury groups with and without transplants had high variations in lesion and engraftment, therefore warranted a larger sample size.

### Unilateral pTBI surgery

Sprague Dawley rats were anesthetized using 2–5% isoflurane in a mixture of 70% nitrous oxide and 30% oxygen. The body temperature was maintained at a normothermic (37°C ± 1°C) level throughout the pTBI via a homeothermic heating pad. The head was secured in the stereotaxic device for insertion of the pTBI probe. We previously described this apparatus which includes a penetrating probe (Kadence Science, Lake Success, NY), a stereotaxic frame (Kopf, Tujunga, CA) equipped with a specially designed probe holder, and a hydraulic pressure-pulse generator (4B080; MITRE, MA). The probe is made of a 20-gauge stainless steel tube with fixed perforations along one end sealed by a piece of airtight elastic tubing. This probe is secured on the probe holder with the unperforated end attached to the pulse generator angled at 50° from the vertical axis and 25° counterclockwise from the midline. Post midline scalp incision, a burr hole (diameter = 4 mm) was made using a dental drill to expose the right frontal pole at +4.5 mm anterior-posterior (AP), +2 mm medial-lateral (ML) to bregma as previously described. Additional bone was removed anterior to the burr hole with the drill to allow insertion of the pTBI probe [5, 7, 10].

The pTBI probe advanced into the cranial window into the right hemisphere to a depth of 12 mm from the surface of the brain. The pulse generator was activated as the probe was in place via a computer. A pressure pulse was calibrated to stimulate a rapid expansion of the water-filled elastic tubing was activated to induce an elliptical-shaped balloon (diameter = 0.633 mm, duration = 40 msec) to a volume 10% of the total brain volume. The inflation/deflation was rapid in order to mimic the generation of a ballistic force shock wave, generating a temporary unilateral cavity in the brain. After the probe was deflated, the probe was immediately removed from the brain and the skin incision was closed with wound clips. The sham surgery included a midline scalp incision, the right frontal burr hole, and the skin incision closed with wound clips [10]. The lesion size stretched from +3.00 to -3.00 mm from bregma [23].

### Human neural stem cell injections

One week post injury, animals were anesthetized again for transplantation. The sham and low dose groups received 0.16 million green fluorescent protein (GFP) expressing human fetal neural stem cells (hNSCs) in Hibernate^®^ medium (BrainBits^®^ LLC). A 50,000 cells/μL suspension was administered via a gas tight 250-μL Hamilton syringe. The high dose group underwent the same procedure but received 1.6 million hNSCs. The cell-filled micro syringe was aligned +2.72 mm AP and +1.5 mm ML (from bregma) for injection into the penumbra. The injection was inserted ventrally to a 6 mm depth. At this location, the first cell drop was administered. After, the injection was raised to a 4 mm depth and the second cell drop was administered. The cell drops were deposited at the following bregma coordinates: +3.5 mm ML at these two depths and -2.28 mm AP with similar ML.

### Histopathology and immunohistochemistry

**Fig 1:**
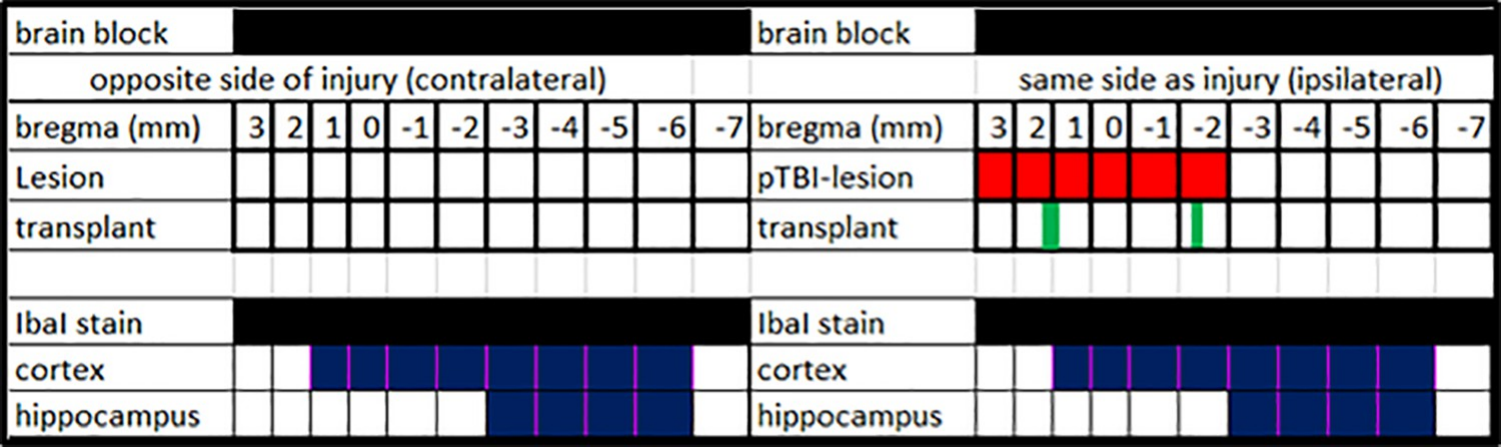
Schematic diagram of a saggital brain section to illustrate the bregma range of the lesion and sites examined. The schematic shows a sagittal view of the 11mm brain block (top row) to provide the context for the lesion, transplant locations with respect to bregma. Next set of rows lists sites examined for microglial morphology. The first column lists names of the rows and subsequent 11 columns represent 11 sections 1 mm apart along rostro caudal axis with features such as lesion and transplant. The rostro caudal axis with respect to bregma (first row) controls for location. The pTBI lesion (filled red squares) in next row is present only on one side of the brain and across 6mm (+3.0mm to -2mm). The transplants (green bars) in row below show locations of the hNSC deposits. In the next set the top row show brain sections stained with IbaI (filled black squares), the sites at which the microglia morphology was examined which includes penumbra regions with spared cortex (blue filled squares) and hippocampus (blue filled squares).

Three months post injury, rats were anesthetized with isoflurane and perfused with 4% paraformaldehyde as previously described [9, 18]. This time-point was selected to allow sufficient time for differentiation of human NSC (NSI-566 RSC) into neuronal phenotypes to enable examination of the relationship between neuroprotection and transplant maturation. After post-fixation, the brains were transferred to a phosphate buffer saline (pH = 7.4) containing 20% sucrose and stored at 4°C. Brains were shipped to FD Neurotechologies, Inc (Columbia, MD) for sectioning. Free floating serial coronal sections (35um) were cut from +1.50 to -7.00 mm from bregma. For analysis, 22 serial sections were produced, each 0.5 mm apart spanning both the lesion and the transplant and stored in free-floating cryopreserve prior to immunostaining. Brain sections were stained with Hematoxylin and Eosin (H&E) and a separate serial group was stained with Iba1 by FD Neurotechnologies, Inc. (Columbia, MD). For Iba1 staining, an ionized calcium-binding adapter molecule 1 (Iba1) 1:400 and a secondary antibody of goat anti-rabbit 594 (Life Technologies; 1:250) were used. Sections were also incubated with 2-(4-aminophenyl)-1H-indole-6-carboxamidine (DAPI) prior to mounting and imaging. Sections were mounted on gelatin coated slides, cover slipped and then shipped back to us for analysis. A schematic diagram is presented to provide context for the lesion, transplant locations with respect to bregma as well as the microglial morphology assessment (Fig 1).

### Image acquisition

The images were acquired using an Olympus Confocal Microscope Fluoview (FV1000) with the following settings (a) z-scan thickness 14 μm thick slides, (b) magnification of 60x, (c) area of 417 x 417 μm, (d) resolution of 1024 x 1024 pixels, (e) multi-area time lapse scans of 3 x 3, and (f) digital 2x zoom. The ipsilateral and contralateral cerebral cortex included the first sections of slides 3–10 i.e., +1.50 mm through -5.78 mm bregma, for a total of 256 images, respectively. The ipsilateral and contralateral dentate gyrus included the first section of slides 7–10 i.e., -2.28 mm to -5.78 mm through with a total of 126 images, respectively. A total of 764 images were taken. These images were saved as an Olympus file type. The regions of interest were selected to compare regions close to the injury site (i.e., ipsilateral cortex, ipsilateral dentate gyrus) to those further from the injury site (i.e., contralateral cortex, contralateral dentate gyrus).

To measure contusion volumes and GFP volume, we used similar approaches previously described [19, 23]. Since the pTBI porencephalic cyst intersects with the lateral ventricle across the rostrocaudal axis of the brain, lesion area was defined as the area of expanded ventricle plus lesion (cyst) minus the area of contralateral ventricle expressed as percent of left hemisphere. For hNSC implant GFP engraftment volume analysis, the % of the left hemisphere was calculated for both the low and high hNSC transplants [22].

### Imaris analysis

**Fig 2:**
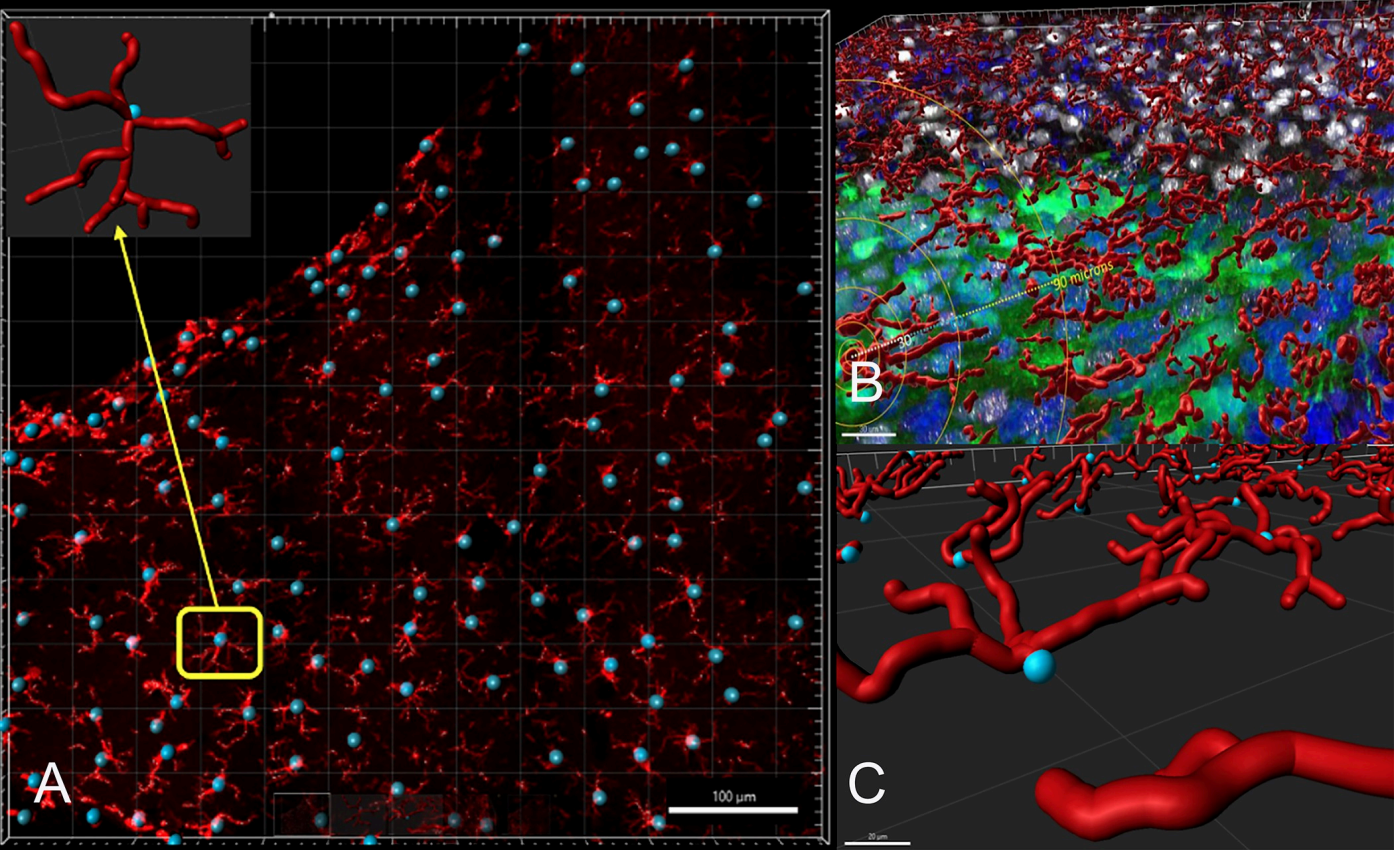
Filament reconstruction. Filament reconstruction was utilized to render a microglia structure. The blue sphere indicated the soma of the microglia. The seed points were connected to create red filaments of varying length. The sphere and filaments were connected to form entire microglia (A). This procedure was performed utilizing Filament Tracer Module in Imaris Software. Magnified representations of the microglia are shown on the right panel with (B) and without (C) fluorescence from other channels. These images correspond to the ipsilateral cortex.

We first used an Imaris 9.7.1 file converter to change the Olympus file type (OIB) to an Imaris file type (IMS) for access in Imaris 9.7.1. The images contained DAPI, Iba1, and GFP channels. In Imaris, Filament Tracer Module was used to reconstruct the microglia cells. The Iba1 channel was selected as the primary channel for Iba1 microglia activation analysis. The other channels were deselected prior to filament reconstruction. A background subtraction, performed on each image, amplified the main Iba1 signals and reduced background fluorescence. The green filament icon helped illustrate the projecting filaments from the soma. The smallest microglia recorded had a 1 μm diameter soma, while the largest microglia had a 14 μm soma due to the level of sections through the cell body. Therefore, the diameter range was standardized across all images (1–14 μm). The spherical blue starting point marked the soma, while the white seed points indicated the filaments projecting from the soma. Imaris automatically identified the microglia’s somas and filaments, and manual adjustments to the starting and seed points ensured the inclusion of all microglia cells for a given image. All of the microglia present in an image were included and no somas were excluded. Imaris connected the starting and seed points, ultimately rendering three-dimensional red microglia. Each Imaris image was analyzed in its entirety via this filament reconstruction procedure. For visual representation, the cylindrical view was selected (Fig 2).

### Quantitative and qualitative analysis

The data type of interest included the “filament number of Sholl intersections.” Imaris produced an Excel data sheet with the raw data. The procedure employed included sorting the Radii from lowest to highest. The radii represented the radial distance from the soma in microns (μm). The Imaris Excel data sheet consisted of the number of intersections at various radii with the radius value capturing the microglia into different levels of microglia activation. The data acquisition was performed for sections between +1.50 mm to -7.00 mm bregma for the ipsilateral/contralateral cortex and the ipsilateral/contralateral dentate gyrus.

The distribution curve of microglial process length (x-axis = radial distance from soma, 0–12 μm) and the number of such processing intersecting the radii (y-axis = number of intersections) serve as a measure of de-ramification/activation [20]. Larger values for both radii and number of intersections correspond to lower levels of microglia activation, while smaller values for both radii and number of intersections correspond to higher levels of microglia activation. Microglia activation was compared using quantitative data obtained from the following four locations: ipsilateral cerebral cortex (region of neurodegeneration), contralateral cerebral cortex (intact cortex), ipsilateral dentate gyrus (region with predominant injury induced axonal degeneration), and contralateral dentate gyrus (intact axons). Data from the Sholl analysis were also plotted along the rostrocaudal axis (+1.50 mm to -7.00 mm). Peak Sholl values were selected for sham, vehicle, low dose, and high dose groups to compare microglia activation. Additionally, we qualitatively classified microglia into their morphological subtypes via Imaris images.

### Statistical analysis

Statistical analysis was performed using Prism 9 (GraphPad Software, Inc. La Jolla, CA). All measures were expressed as mean ± standard error of the mean (SEM). Dose dependent neuroprotection was compared using a one-way analysis of variance (ANOVA) followed by Dunnett’s multiple comparisons *post hoc* test. Rostrocaudal axis results were compared using repeated measures analysis of variance (ANOVA) followed by Tukey’s multiple comparisons *post hoc* test. Correlation analysis was performed comparing lesion volume to dose.

## Results

### Lesion volume is inversely related to hNSCs dosage (GFP engraftment)

**Fig 3:**
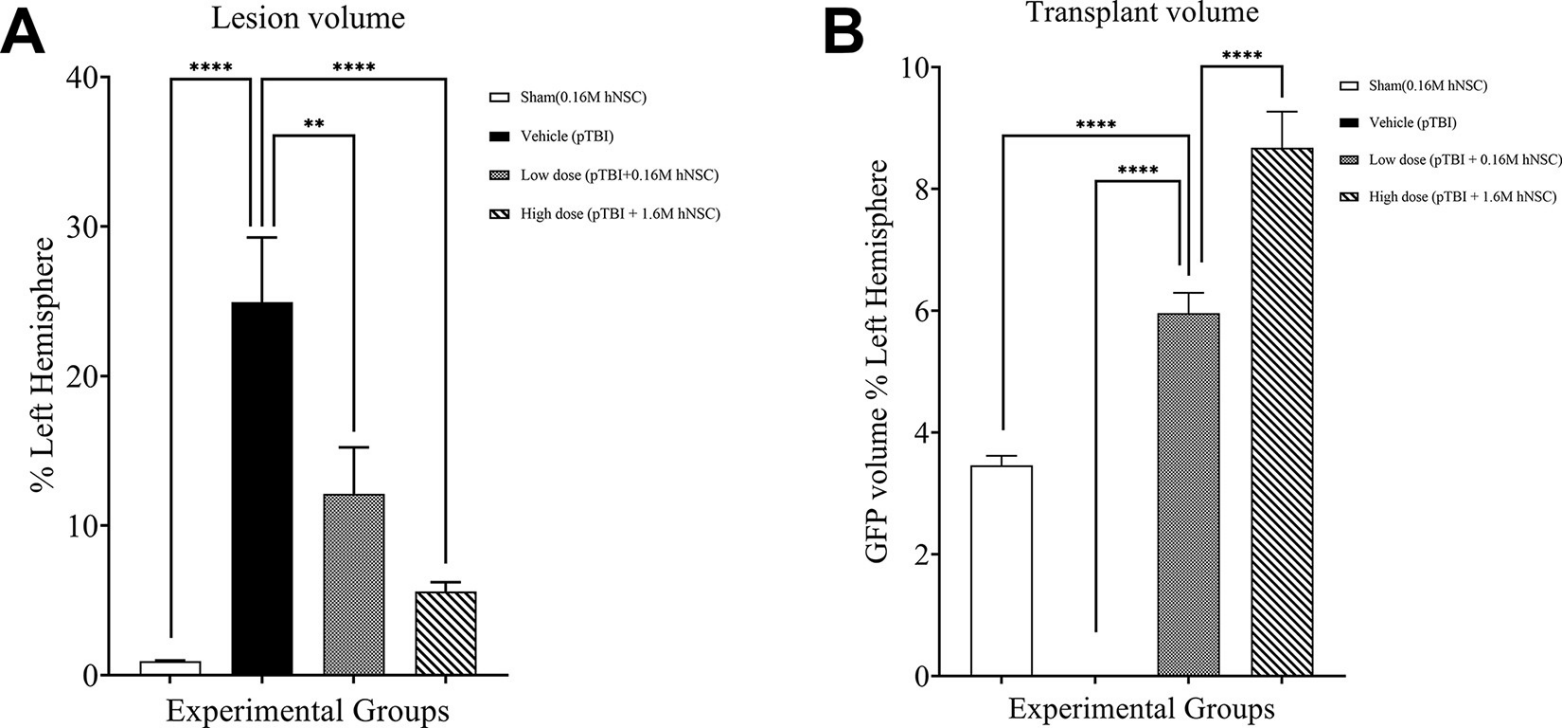
Cell dose dependent mitigation of pTBI lesion progression. (A) The mean lesion volume (±SEM) as a % of the left hemisphere shows the injury effect and hNSC neuroprotection. Statistical significance determined using a one-way ANOVA followed by a Dunnett’s *post hoc* test. (B) The GFP volume as a % of the left hemisphere captures dose response with the high dose group higher than the low dose group and sham group. Statistical significance was similarly determined using a one-way ANOVA followed by a Dunnett’s *post hoc* test. ****p < 0.0001; **p < 0.01.

Plotting mean lesion volume (Fig 3A) and extent of engraftment (Fig 3B) shows the inverse relationship between lesion volume and hNSCs dosage or GFP engraftment. Statistical significance was determined with a one-way ANOVA for Lesion % left hemisphere (LH), *F(*3, 34) = 16.68. This was followed by a Dunnett’s *post hoc* test which captured the injury effect: (i) vehicle vs sham *p* < 0.0001, (ii) vehicle vs low dose *p* < 0.01, (iii) vehicle vs high dose *p* < 0.0001. Similarly, statistical significance was determined using a one-way ANOVA for GFP Volume % LH, *F*(3, 34) = 104.4. This was also followed by Dunnett’s *post hoc* test which captured the therapeutic effect: (i) low dose vs sham *p* < 0.0001, (ii) low dose vs vehicle *p* < 0.0001, (iii) low dose vs high dose *p* < 0.0001. Correlation analysis of lesion volume versus dose was also significant (p = 0.0374, r = -0.3532. However, there was no correlation between lesion volume and transplant volume (*p* = 0.717, r = 0.07482).

### Morphological classification of microglia subtypes captures injury and therapeutic effects

**Fig 4:**
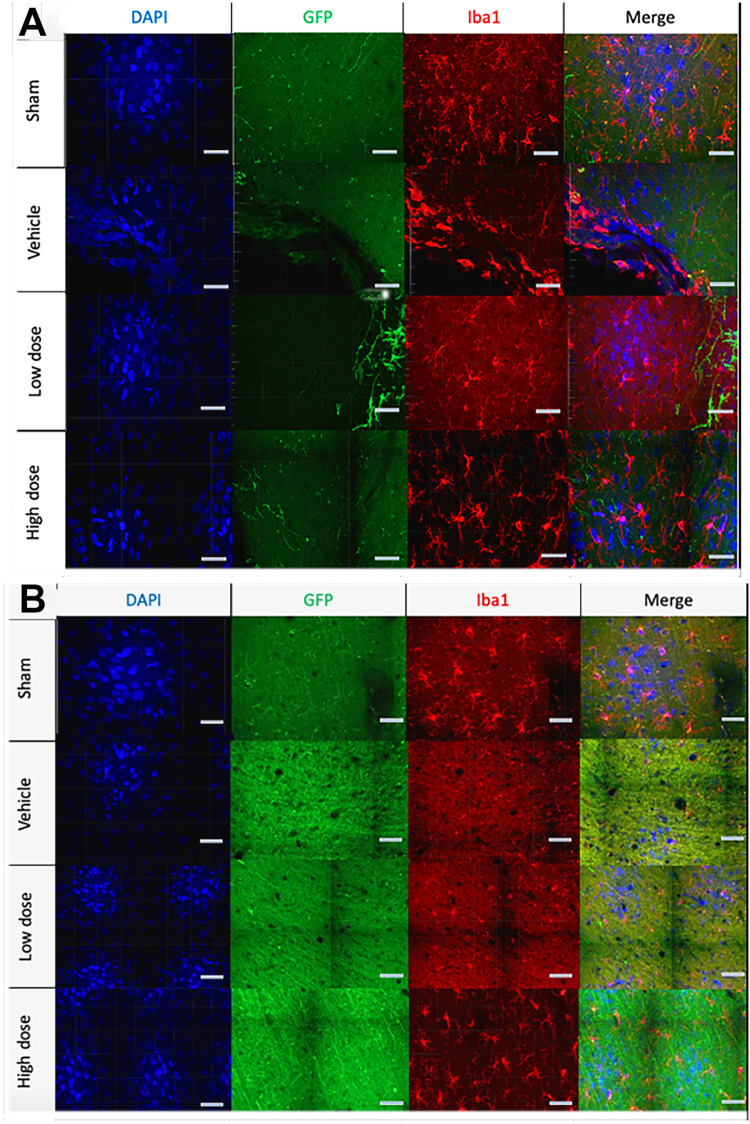
High power images for morphological analysis. High power images of (A) ipsilateral cortex and (B) ipsilateral dentate gyrus from four experimental groups: Sham (first), Vehicle (second), low dose (third) and high dose (fourth rows) in channels (labeled above the image) shows activated morphology (rounded microglia without processes), ramified microglia (highly branched). The merge column indicates evidence for the degree of microglia activation. Scale bar is 50 μm.

**Fig 5:**
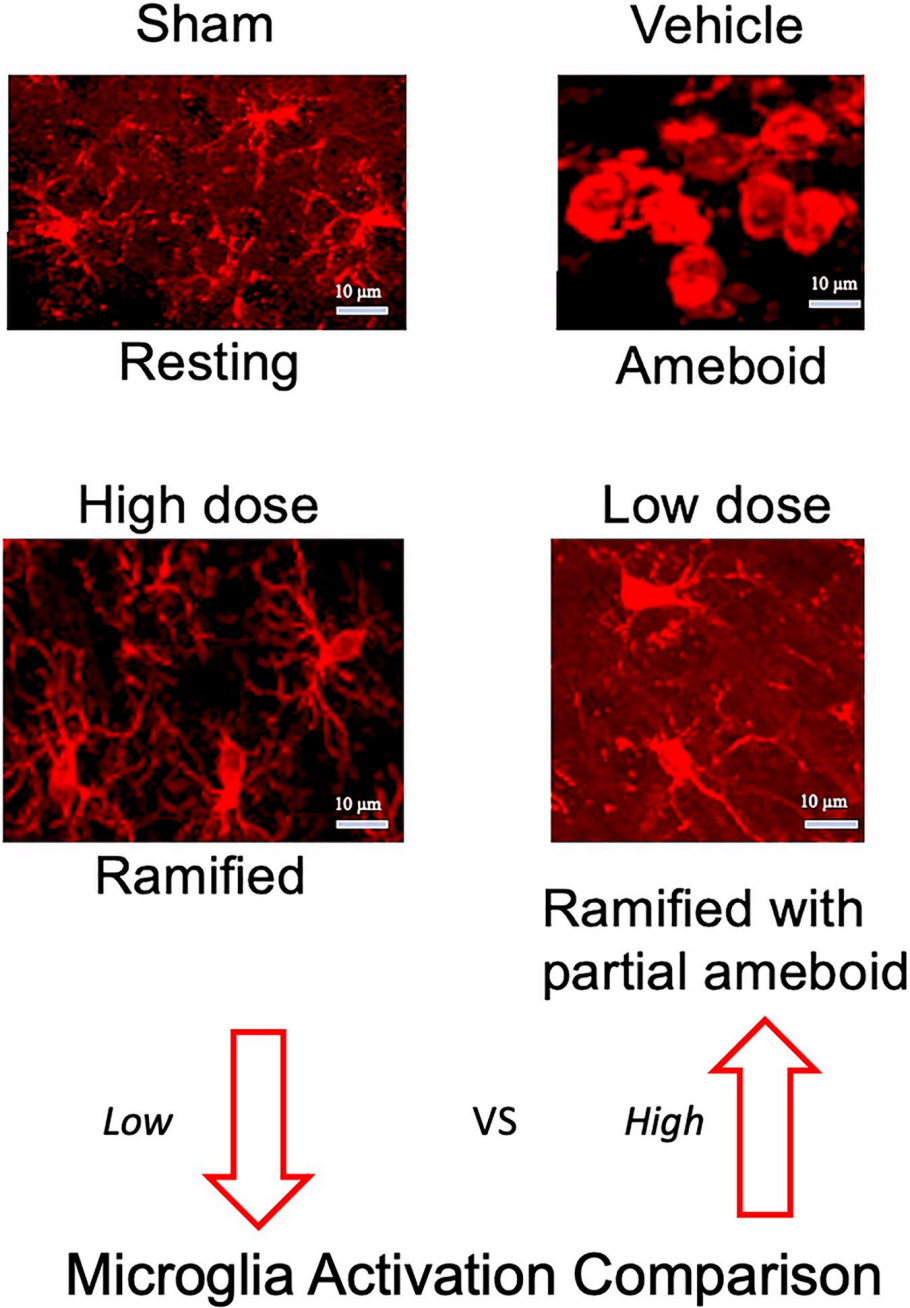
Microglia morphological transition. The microglia activation transition can be represented from lowest to highest microglia activation corresponding to the analyzed microglia morphology: sham (resting) < high dose (ramified)< low dose (ramified with partial ameboid) < vehicle (ameboid).

Qualitative morphological assessment of the various experimental groups demonstrated the injury effect. In the ipsilateral/contralateral cortex and the ipsilateral/contralateral dentate gyrus, vehicle microglia adopted ameboid morphology with shorter and diminished filaments, while the sham microglia were ‘resting’ with longer and extended filaments. The therapeutic effect was also evident. In the ipsilateral cortex (Fig 4A) and dentate gyrus (Fig 4B) the vehicle microglia appeared ameboid, while the low dose had ramified with partial ameboid features, in contrast the microglia in high dose transplant group sections were predominantly ramified. Contralateral structures are not shown. The overall microglia morphological transition from low to high microglia activation: sham (resting), high dose (ramified), low dose (ramified with partial ameboid), and vehicle (ameboid) (Fig 5).

### Microglia activation among dosage types vary along the rostrocaudal axis

**Fig 6:**
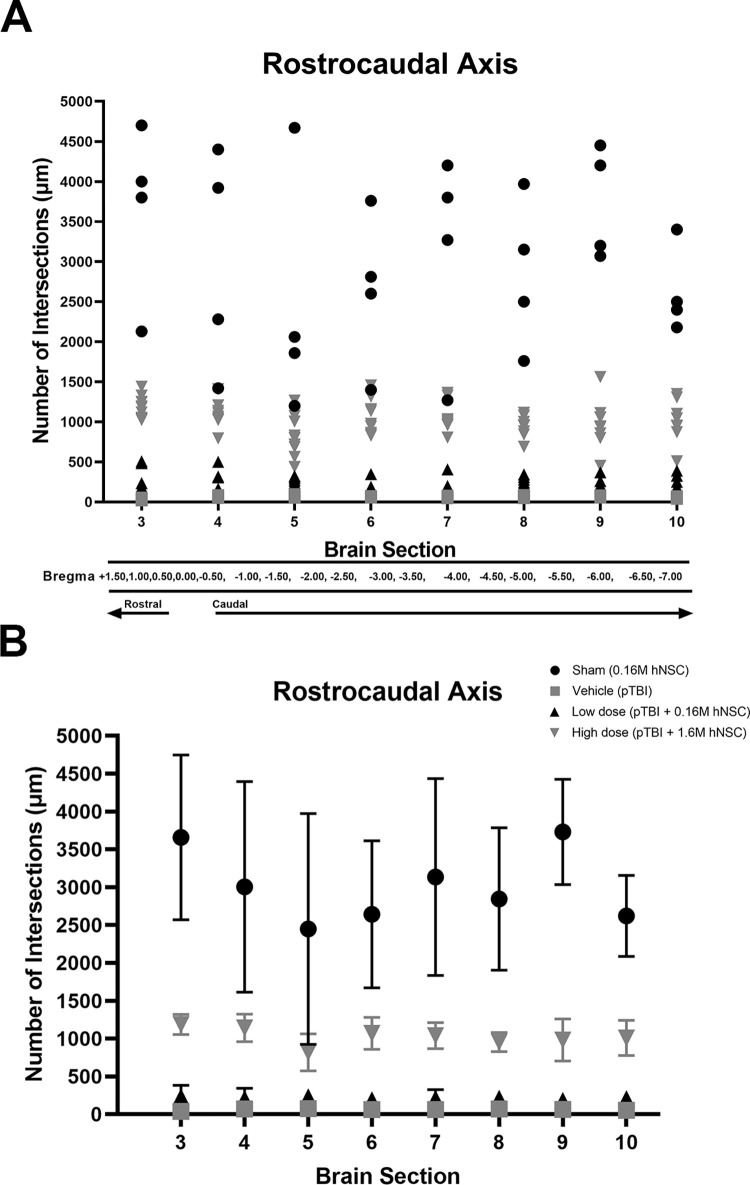
Rostrocaudal axis. (A) A scatter plot of individuals from experimental groups along the rostrocaudal axis shows the extent of injury and location of therapeutic effect in penumbra. The number of intersections trends higher in Sham and lower in Vehicle along the rostrocaudal axis, with low dose and high dose between them. Peak radius intersections (low microglia activation) was similar to Sham only in the penumbra (-2.5 mm to -3.5 mm bregma) of high dose while at the lesion core (-0.5 mm to -2.0 mm bregma) values were still low (activated microglia). (B) Along the rostrocaudal axis, statistical significance was determined for the mean number of intersections (+SEM) using a repeated measures ANOVA followed by Tukey’s *post hoc* test.

The distribution of filament lengths peaks at distinct Sholl radii for homeostatic and proinflammatory activated microglia. In order to quantitatively compare microglia morphology (a measure of proinflammatory activation) across the rostrocaudal axis and between experimental groups, microglia filament lengths in images at aforementioned sites in various experimental groups were plotted at peak Sholl radius as number of intersections. Filament length was calculated from the center of the microglia soma (0 μm) extending radially out. The intersections are plotted on the y-axis and brain section on the x-axis (+1.50 mm to -7.00 mm) in Fig 6. The sham operated group had the highest number of intersections, ranging from ~1250–4700 μm consistent with homeostatic microglia morphology/low activation. In contrast, vehicle treated pTBI animals had the lowest number of intersections ranging from ~100–150 μm similar to previous observations of high levels of microglia activation. Compared to the vehicle pTBI, values for the low dose of hNSCs ranged from ~250–500 μm whereas the high dose ranged from ~500–1750 μm. These increased values in high dose group are suggestive of decreased microglia activation compared to vehicle group (Fig 6A). Results from a repeated measures ANOVA indicated statistical significance in the number of intersections along the rostrocaudal axis *F*(3, 252) = 467.5, *p* < .0001. This was followed by a Tukey’s *post hoc* test: (i) sham vs vehicle *p* < 0.0001, (ii) sham vs low dose *p* < 0.0001, (iii) sham vs high dose *p* < 0.0001, (iv) vehicle vs low dose *p* < .1322 (ns), (v) vehicle vs low dose *p* < 0.0001, (vi) low dose vs high dose *p* < .0001 (Fig 6B).

### Sholl analysis assessment of microglia activation captures injury and therapeutic effects

**Fig 7:**
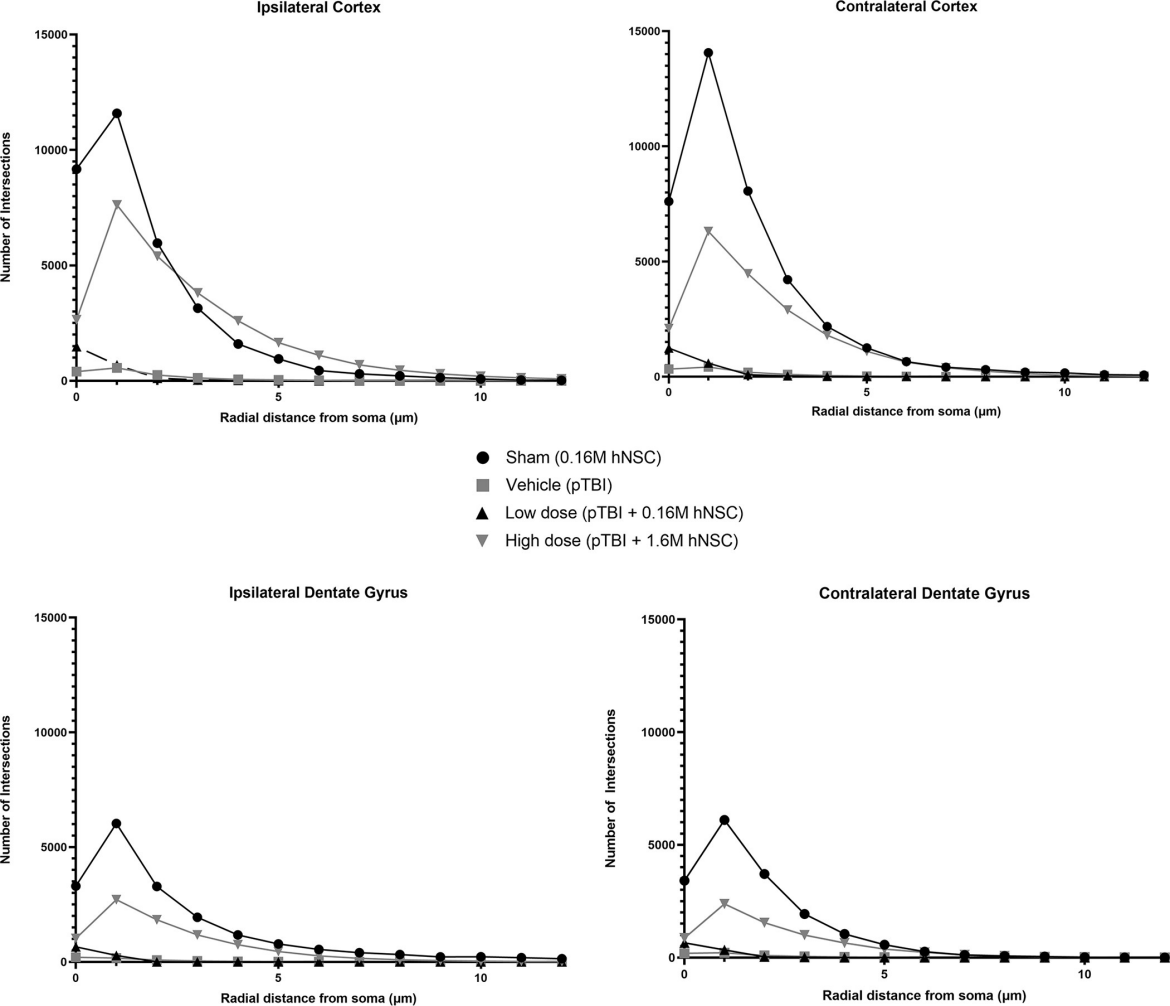
Sholl analysis of distance from soma graphs. (A) Ipsilateral Cortex Peak Sham Intersections: (~12,000 intersections), Vehicle (~500 intersections), Low dose (~1,000 intersections), High dose (~7,500 intersections). (B) Contralateral Cortex Peak Intersections: Sham (~14,000 intersections), Vehicle (~500 intersections), Low dose (~750 intersections), High dose (~6,500 intersections). (C) Ipsilateral Dentate Gyrus Peak Intersections: Sham (~6,500 intersections), Vehicle (~250 intersections), Low dose (~550 intersections), High dose (~3,000 intersections). (D) Contralateral Dentate Gyrus Peak Intersections: Sham (~6,500 intersections), Vehicle (~250 intersections), Low dose (~550 intersections), High dose (~2,500 intersections).

Quantitative Sholl analysis again demonstrated pTBI and hNSC transplantation effects. Comparing each location, the shorter and diminished peak of intersections for the pTBI vehicle compared to the longer processes of sham, is indicative of the injury effect. In the ipsilateral cortex, the vehicle had ~500 intersections, while the sham had ~12,000 intersections (Fig 7A). In the contralateral cortex, the pTBI vehicle had ~500 intersections, while the sham operated had ~14,000 (Fig 7B). In the ipsilateral dentate gyrus, the vehicle pTBI had ~250 intersections, while the sham operated had 6,500 intersections (Fig 7C). In the contralateral dentate gyrus, the vehicle pTBI also had ~250 intersections, while the sham had ~6,500 intersections (Fig 7D). Comparing each location, the shorter and diminished peak of intersections for the pTBI vehicle compared to the longer processes in the low dose and high dose, suggests a therapeutic effect.

## Discussion

The transplantation of NSCs as a strategy to repair the damaged brain is currently being studied in both experimental and clinical studies [4]. In the area of neuroprotection, recent studies have also reported location-dependent benefits of hNSC transplantation after pTBI when cells were transplanted 1 week after injury [19]. This clinically relevant TBI model has been reported to show evidence for progressive degeneration associated with prolonged microglia/macrophage activation. Lee and colleagues [9] reported following pTBI, increased levels of proinflammatory cytokines together with morphological evidence for prolonged microglial activation. Because the dose (number of cells) may be an important factor in determining the benefits of hNSC transplantation, the current study was conducted to evaluate for the first time the dose-dependent hNSC effects on patterns of chronic microglial activation. For this goal, we used a series of morphological quantification techniques to investigate the arborization patterns of microglia in several ipsilateral and contralateral brain structures.

We demonstrated a hNSC dose-response effect on lesion volume at 3 months after pTBI. Both low (0.16 million) and high (1.6 million) hNSC doses significantly reduced lesion volumes compared to vehicle pTBI. Although the higher dose provided a trend toward better protection, there was no significant difference between the 2 treatment pTBI groups. However, in terms of transplant volumes at 3 months, the higher dose of hNSC produced a significantly higher volume of GFP positive cells compared to the lower hNSC dose at 3 months as expected. The fact that lesion volume was related to hNSCs volume may indicate a need for larger numbers of hNSC to be implanted to provide optimal conditions for long term protection. However, lesion volume was not related to transplant volume indicating that the transplant did not have a measurable impact on lesion volume.

Qualitative immunocytochemical assessment of the various experimental groups demonstrated regional patterns of microglial activation after pTBI compared to sham operated animals. In the ipsilateral cortex and the ipsilateral dentate gyrus, vehicle pTBI microglia adopted ameboid morphology with short and diminished filaments, while the sham operated microglia were ‘resting’ with longer and extended filaments. The therapeutic effect of hNSC transplantation was also demonstrated. In the ipsilateral cortex and dentate gyrus, the vehicle pTBI microglia appeared ameboid compared to ramified morphology on the contralateral side. The low hNSC dose showed microglia with a range of ramified and ameboid processes. In contrast the high hNSC dose contained mostly ramified microglia. The overall microglia morphology transitioned from low to high microglia activation: sham (resting), high dose (ramified), low dose (ramified with partial ameboid), and vehicle (ameboid).

Using the Sholl analysis for the first time after experimental pTBI, we provided evidence for pTBI leading to chronic patterns of microglia activation indicated by the number of intersections along the neuroaxis. Compared to sham operated animals that received 0.16 million hNSCs significant reductions in peak values of intersections were demonstrated in the low dose and vehicle treated pTBI groups. The data plotted along the rostrocaudal axis demonstrates the injury effect. Interestingly, some evidence for increased microglial activation was seen in all brain regions using Sholl analysis analyzed including the ipsilateral and contralateral cerebral cortex and dentate gyrus. In each brain region, vehicle pTBI group consistently showed the lowest numbers of intersections especially in the region of the cell soma. Taken together, these results indicate that this model of pTBI has bilateral effects on the inflammatory responses to both cortical and subcortical regions.

In the current study we also demonstrated a treatment effect specifically in the high dose hNSC transplantation group. Across the bregma levels, the pTBI vehicle and low dose pTBI had diminished numbers of intersections. In contrast, sham operated animals showed the highest numbers of intersections while values for the low dose pTBI were between the vehicle TBI and high hNSC dose animal groups. The primary finding indicates that the high dose of 1.6 million hNSCs correlated with decreased microglia activation, while the low dose of 0.16 million hNSCs correlated with higher microglia activation.

Our findings are consistent with the results of Lee and colleagues [9] who reported the appearance of activated ameboid microglia primarily in the perilesional cerebral cortex using routine Iba1 immunocytochemistry at 24 hrs after pTBI. In the current study using Imaris software for the Sholl analysis approach, we also found evidence for microglia activation in the ipsilateral cortex near the injury site as well in more remote areas at 3 months after pTBI. In ipsilateral cortical and dentate gyrus regions after injury, the number of intersections were severely reduced in vehicle treated pTBI animals versus sham operated controls. This was most apparent in regions near the cell body ranging from 0 to 5 um. The fact that pTBI produced widespread microglial activation for a prolonged period after injury emphasizes the importance of this inflammatory response in secondary injury processes.

The potential for hNSCs to protect the adult brain after acute or more progressive injuries has been previously reported [19, 24–27]. In a study by Soares and McIntosh [25], fetal cortical tissue was injected into the injured brain at various periods after TBI. Interestingly, NSC injections at 2 days, 1 week or 2 weeks demonstrated transplant survival and incorporation with the host brain and attenuated glial scarring. In animals that were injected 2 days after TBI, neuronal sparing of CA2/CA3 hippocampal regions was also noted. In a more recent publication, Hu and colleagues [19] reported a location-dependent neuroprotective effect of hNSC transplantation when cells were injected 1-week after pTBI. Interestingly, evidence for cortical tissue sparing at 12 weeks was reported when cell transplants were placed in the tissue surrounding the lesion but not when cells were injected directly into the lesion core.

Several mechanisms have been suggested for the neuroprotective effects of hNSCs including reducing secondary inflammatory events as well as the synthesis and release of neurotrophic factors or therapeutic gene products [19, 28–30]. Recent evidence suggests that NSC transplantation is an effective therapy for acute brain injury through multiple mechanisms such as preservation of the blood-brain barrier, alleviation of neuroinflammation and enhanced neurogenesis [30–32]. In the study by Peruzzotti-Jametti and colleagues [29], an anti-inflammatory mechanism was demonstrated where NSCs reduced succinate levels thereby decreasing mononuclear phagocyte infiltration and secondary damage. In the current study, hNSC transplantation may therefore attenuate the widespread activation of microglia/macrophages and increased tissue sparing in perilesional areas by multiple mechanisms.

Future studies are needed before hNSCs can be utilized to treat patients suffering from TBI and from the secondary damaging effects of microglia activation. These studies may include replication of these dosage quantities with the inclusion of more dosages to verify if 1.6 million stem cells is the optimal dosage to reduce microglia activation and promote behavioral recovery. The practical implications of this study include the potential of hNSCs being utilized as a treatment in pTBI patients to combat the harmful secondary effects of microglia activation. The findings from both the quantitative Sholl analysis and the qualitative morphological analysis similarly demonstrated that the 1.6 million hNSCs have an effect on lesion volume and widespread patterns of microglial activation.

In summary, the current findings demonstrate a non-biased quantitative strategy for providing evidence for microglial activation in multiple brain regions after pTBI. Previous studies have primarily implemented a qualitative morphological approach to characterizing microglia activation post TBI. The Sholl analysis approach offers a different, yet innovative and robust strategy to assess microglia activation in TBI models where the effects of various treatment strategies on structural indicators of cellular activation can be assessed. Also, our results provide new information regarding dose response effects of hNSC administered in terms of altering chronic lesion volume and widespread inflammatory processes. Future studies may be employed to confirm the best dosage of hNSCs for utilization as a potential treatment for TBI leading to reduced structural and neurological deficits.
